# Rheumatic Pneumonia

**Published:** 1855-11

**Authors:** 


Rheumatic Pneumonia.—About a month since a young man was admitted with all the signs of pneumonia, and, kermes having been administered, the next day all traces of the pneumonia had disappeared. To what were we to attribute so sudden a retrocession? Was it the result of treatment, or must we seek for the cause in some peculiarities attaching to the nature of the disease itself? The latter interpretation received some light from what was observed at the next visit, when the left great toe was found red, swollen and painful, the tendinous sheaths along the dorsum of the foot exhibited a like condition. Next day the right foot was similarly affected, though in a less degree. Two days ago a woman was admitted with the following symptoms: strong febrile action, redness and swelling of the left leg and foot, and severe pain in the entire upper extremity and trunk of the same side, the pain exciting cries on moving the parts. The patient especially suffered at the left side of the chest, but no abnormal sounds were audible. During the night cough came on, and in the morning a manifest souffle was audible in the supra spinal fossa, while around and in the infraspinal fossa was heard a fine subcrepitant râle. During the cough dry crepitating râle and bronchophony were heard, and two or three pneumonic sputa were expelled. This morning all signs of pneumonia have vanished. Here again I hesitate to attribute such prompt resolution to the treatment, especially as the apex was the part involved—a form of pneumonia regarded by all physicians as especially serious. I prefer explaining so rapid a termination by the nature of the pneumonia itself, which I regard as *rheumatic*.

Too partial to localization, practitioners are only accustomed to recognize rheumatism as affecting certain tissues, namely, the muscles, the aponeuroses, and the joints, and when it manifests itself elsewhere they call it by some other name. This is as if we only acknowledge syphilis as we observe it on the penis, and made so many distinct affections of its manifestations on the throat, skin, etc. But syphilis is recognized to be the disease in all these accidents, and why should it not be so with rheumatism? That it attacks all serous membranes is an indisputable fact since Bouillaud's beautiful researches, which have so much advanced the pathology of the heart. When in the course of acute articular rheumatism any of the serous membranes become affected, it is termed a pericarditis, pleuritis, meningisis, etc., according to the membrane attacked. This is right enough so far as it goes; but for the proper denomination of the disease, which is a kind of definition in a single word, we ought to add the epithet "rheumatic." When a man accustomed to suffer from rheumatism acquires, as a consequence of cold, a pain of the shoulder, hip, etc., he at once says he has an attack of rheumatism. But instead of this pain let there be a sore throat, and both patient and doctor cease to be logical, and call it angina instead of rheumatism; just if there were not a true rheumatic pain of the fibrous parts of the pharynx and palate, pain followed by fluxion, tumefaction, and redness of the pharyngeal mucous membrane. Do we not find rheumatism of the fibro-serous tissues of a joint accompanied by tumefaction of the subcutaneous cellular tissue, and bright redness of the skin; and why should we not admit the same influence in the delicate and vascular mucous membrane? For my part I should not hesitate to recognize a rheumatism in such a case, or, if you like it better, a rheumatic angina.

This distinction may serve for the explanation of the very great differences observed in the progress and termination of anginas, regarded by some physicians as being of the same nature. Thus, simple inflammation of the tonsils goes through all its stages, in spite of whatever treatment may be opposed to it, and a patient accustomed to such attacks will warn his attendants of the inutility of endeavoring to prevent the formation of abscess. A rheumatic angina, on the contrary, will often disappear in the course of a night, whatever the treatment adopted, leaving the physician astonished at his therapeutical success, the result, however, being really due to the essentially mobile character of the affection. Descending lower down in the digestive canal, we can explain those sudden diarrhœas which manifest themselves under the influence of a chill. The fibrous portions of the canal become painful, and the contractions more considerable and more frequent, a fluxionary movement being at the same time established towards the mucous membrane, the secretions of which are increased. Such diarrhœas are of short duration, unless, indeed, the rheumatism takes on, as it may anywhere, a chronic character.

After these considerations, does it seem strange to admit a rheumatic pneumonia? Suppose the pulmonary tissue, or what is the same thing, the fibrous tissue of the extremity of the bronchi, becomes seized with rheumatism, what are the immediate results? Tumefaction and congestion of the mucous membrane and an infiltration of the cellular tissue; that is to say, the anatomo-pathological conditions of œdema or of pneumonia in its earliest stage; with this peculiarity, that such lesions, participating in the fugacious nature of rheumatism, do not possess the fixity and persistence of the lesions of ordinary pneumonia. It is in cases like these that therapeutical results seem marvelous, and so they would in our own two cases had we not a better reason to give for the rapidity of the cure. They were in fact the examples of rheumatic pneumonia, the one occurring in a young man who was at the same time suffering from rheumatism of both feet, and the other in a girl who had formerly had rheumatism, and, together with the pain in the chest, complained of rheumatic pains along the whole of the same side of the body. In similar cases, I shall not hesitate to admit the existence of rheumatic pneumonia, too happy only thus to complete my diagnosis, and to become enlightened as to the amount of importance that should properly attach to my therapeutics.—*London Lancet.*

